# Clinical relevance of IgE-antibodies to PR-10 proteins in patients with syndrome of oral allergy

**DOI:** 10.1186/2045-7022-3-S3-P90

**Published:** 2013-07-25

**Authors:** E Bass, M Mokronosova

**Affiliations:** 1 Mechnikov’s Research Institute for Vaccines and Sera, Moscow, Russian Federation

## Background

A considerable number of pollen-allergic patients develops allergy to plant foods, which has been attributed to cross-reactivity between food and pollen allergens belongs to PR-10 proteins. The majority of these patients refuse from take the foods containing PR-10 explaining bad taste or sense of itch in the mouth - syndrome of oral allergy (SOA). It is assumed that the primary is sensitization to pollen origin PR-10 proteins and induction of the IgE-aB to the fruit & vegetable origin PR-10 is secondary. The aim of the study: to measure the IgE-aB to PR-10 proteins in patients with SOA.

## Methods

There were 21 patients (pts) with SOA (12 male/9 female, age 1 – 42 years) were observed. From them 18 was suffered from pollen allergy. Component-resolved diagnosis was provided by ImmunoCap ISAC (ThermoFisher Scientific, Sweden) with all 21 sera of observed pts.

## Results

18/21 of pts had positive levels of specific IgE-aB to pollen PR-10 allergens (rAln g 1, rBet v 1 and rCor a 1.0101). The levels of specific IgE-aB to rBet v 1 were positive in 17/21 pts (81%); to rAln g 1 in 12/21 (57,1%); to rCor a 1.0101 in 8/21 (38,1%). There were 17/21 pts who had specific IgE-aB both to pollen and to the food allergens. The levels of specific IgE-aB to the apple (rMal d 1) were positive in 17/21 cases (81%); to peach (rPru p 1) and peanut (rAra h 8) in 13/21 (69%); to hazelnut (rCor a 1.0401) and soybeans (rGly m 4) in 11/21 (52,4%); to the carrot (rDau c 1) and celery (rApi g 1) in 3/21 (14,3%) cases; to rAct d 8 (kiwi) were positive 2/21 (9,5%) of pts. The SOA to such fruits as apple, peach and hazelnut is often in pts with SOA. Clinical manifestation of allergy to peanut and soybeans are rarer. There were IgE-aB only to the plant food PR-10 proteins but not to the pollen in 3/21 samples of sera (14, 3%). One of these pts had specific IgE-aB only to rMal d 1 (apple), one – to rAra h 8 (peanut), and one was sensitized to the apple, hazelnut and soybeans.

## Conclusion

The sensitization to PR-10 protein is followed by primary immune response to tree’s pollen, predominantly rBet v 1. But in some cases the primary sensitization to PR-10 was realized to plant foods. The SOA develops in sensitized patients after eating nuts, fruits and vegetables (except peanuts and soybeans) containing PR-10 proteins.

## Disclosure of interest

None declared.

